# Exploring and Accounting for Genetically Driven Effect Heterogeneity in Mendelian Randomization

**DOI:** 10.1002/gepi.22587

**Published:** 2024-09-22

**Authors:** Annika Jaitner, Krasimira Tsaneva‐Atanasova, Rachel M. Freathy, Jack Bowden

**Affiliations:** ^1^ Department of Clinical and Biomedical Sciences, Faculty of Health and Life Sciences University of Exeter Exeter UK; ^2^ Department of Mathematics and Statistics, Faculty of Environment, Science and Economy University of Exeter Exeter UK; ^3^ EPSRC Hub for Quantitative Modelling in Healthcare University of Exeter Exeter UK; ^4^ Novo Nordisk Research Centre Oxford Oxford UK

**Keywords:** ALSPAC, birth weight, causal inference, heterogeneity, mendelian randomization

## Abstract

Mendelian randomization (MR) is a framework to estimate the causal effect of a modifiable health exposure, drug target or pharmaceutical intervention on a downstream outcome by using genetic variants as instrumental variables. A crucial assumption allowing estimation of the average causal effect in MR, termed *homogeneity*, is that the causal effect does not vary across levels of any instrument used in the analysis. In contrast, the science of pharmacogenetics seeks to actively uncover and exploit genetically driven effect heterogeneity for the purposes of precision medicine. In this study, we consider a recently proposed method for performing pharmacogenetic analysis on observational data—the Triangulation WIthin a STudy (TWIST) framework—and explore how it can be combined with traditional MR approaches to properly characterise average causal effects and genetically driven effect heterogeneity. We propose two new methods which not only estimate the genetically driven effect heterogeneity but also enable the estimation of a causal effect in the genetic group with and without the risk allele separately. Both methods utilise homogeneity‐respecting and homogeneity‐violating genetic variants and rely on a different set of assumptions. Using data from the ALSPAC study, we apply our new methods to estimate the causal effect of smoking before and during pregnancy on offspring birth weight in mothers whose genetics mean they find it (relatively) easier or harder to quit smoking.

## 1 Introduction

Confirming or refuting causal relationships is difficult in observational study settings as one can never be sure if all confounders have been identified, appropriately measured and adjusted for. However, one can take advantage of random genetic inheritance from parents to offspring in an observational analysis to help uncover true causal mechanisms and estimate the causal effect of health interventions (Davey Smith, and Ebrahim 2003). Mendelian randomization (MR) is the formal science of using genetic variants as instrumental variables (IVs) for this purpose (Bowden and Holmes 2019). Rather than testing the direct association between an exposure and outcome, a genetically predicted exposure is used instead. Under the assumption of random distribution of genetic variants from parents to offspring at conception, an individual's genetically predicted exposure should be far less susceptible to confounding bias. MR requires three core assumptions to hold for a genetic variant, G, to be valid instrument to test for a causal relationship between a modifiable exposure and health outcome (Lawlor et al. 2008). These are termed the relevance assumption, the independence assumption and the exclusion restriction. To go beyond testing for causality, an additional assumption is required to estimate (or ‘point identify’) the causal effect. The most commonly used fourth assumption is homogeneity. It states that the causal effect an individual experiences is not affected by the value of their genetic instrument. When this is satisfied, an IV analysis can in theory estimate the average causal effect (ACE) of an intervention on the exposure for the entire study population. However, for continuous outcomes, this assumption is often biologically implausible unless a suitable ‘typical’ range for the exposure is defined (Hernán and Robins 2006). In cases where homogeneity is deemed implausible, an alternative assumption termed monotonicity can instead be applied to enable causal estimation (Bowden et al. 2021). In the context of an MR study using a genetic variant, G, monotonicity means that there is no individual whose exposure would be higher if they did not carry the exposure raising allele of G than if they did. Such individuals would be ‘Defiers’, and assuming that none exist allows the estimation of the causal effect in the subset of ‘Compliers’—defined as the group of individuals whose exposure level would always be greater with the exposure‐raising allele of G than without.

Although homogeneity is typically invoked for interpretation of causal estimates in MR studies, in pharmacogenetic investigations genetic variants are explicitly sought to explain apparent heterogeneity in a treatment's effectiveness. For example, many pharmaceutical interventions are pro‐drugs, which require a specific metabolic process to occur for the patient to experience the full treatment effect. If the patient has a genetic variant that hinders the drug's metabolism (e.g., a ‘Loss‐of‐function’ [LoF] mutation), the treatment effect may be less pronounced in individuals who carry it. For example, Pilling et al. (2021) showed that *CYP2C19* LoF alleles were associated with higher incidence of ischaemic events among those taking the commonly prescribed anti‐stroke drug, Clopidogrel. National Institute of Health and Care Excellence guidance now recommends genotyping individuals on Clopidogrel who experience an ischaemic event, with a view to altering their medication if the LoF variant is found (Advisory Committee of NICE n.d.).

Observational data can be used to quantify the extent of genetically driven treatment effect heterogeneity, but the analysis can be compromised by strong confounding by indication and off‐target genetic effects on the outcome of interest that are independent of any gene–drug interaction. A recently proposed method of pharmacogenetic causal inference using observational data—Triangulation Within a Study (TWIST) (Bowden et al. 2021)—defined the assumptions required to estimate the difference in treatment effect estimates between those with and without a pharmacogenetic variant, as a measure of genetically driven effect heterogeneity. A range of different methods were proposed to estimate this quantity as well as a framework for combining them if sufficiently similar. Although it is a useful tool for estimating this difference, in its most basic form it cannot estimate the causal effect of treatment on the outcome in each genetic group, which is a limitation.

Instances of genetically driven effect heterogeneity do exist in mainstream epidemiological investigations of nonpharmaceutical interventions. For example, smoking in pregnancy has been shown to have measurable consequences on offspring birth weight, which is an important marker of long‐term health (Pereira et al. 2022). Specifically, Freathy et al. (2009) show that single‐nucleotide polymorphism (SNP) rs1051730 on chromosome 15 is associated with smoking cessation during pregnancy as well as smoking quantity. However, the same SNP is not associated with smoking initiation. Therefore, mothers with the rs1051730 risk allele are not more likely to smoke than mothers without, but if they do smoke they tend to smoke more heavily than non‐carriers and find it harder to quit, meaning the effect of smoking on birth weight could easily be moderated by rs1051730.

In this study, we review the standard MR method, which utilises homogeneity‐respecting genetic instruments, and the TWIST method, which utilises homogeneity‐violating instruments. We highlight the different conceptual starting points for each approach, in terms of their modelling assumptions, and how estimates are biased if these assumptions are violated. Subsequently, we explore the integration of both sets of instruments into a unified analysis to properly characterise the ACEs and genetically driven effect heterogeneity. Using data from the ALSPAC study, we apply our new method to estimate the causal effect of smoking on offspring birth weight in distinct genetic subgroups of pregnant mothers; the magnitude of the effect heterogeneity; and the potential public health impact of genetically targeted treatment going forward.

## 2 Methods

Let S and G be binary variables capturing the exposure and genetic variant of interest. In our applied example, S reflects the smoking status of the mother. We allow for the effect of the exposure on the outcome, *Y*, to be altered through an interaction with G, denoted as $S^{*}=G\times S$. To motivate the method, we assume the following linear interaction model for the mean outcome Y given S,G, and additionally measured (Z) and unmeasured (U) confounders of S and Y respectively:

(1) $$\begin{array}{ccc} E\left[Y|S,G,Z,U\right] & = & \gamma_{0}+\beta_{1}SG+\beta_{0}S\left(1-G\right)+\gamma_{YG}G+\gamma_{YZ}Z+\gamma_{YU}U\\ & = & \gamma_{0}+\beta_{0}S+\left(\beta_{1}-\beta_{0}\right)S^{*}+\gamma_{YG}G+\gamma_{YZ}Z+\gamma_{YU}U \end{array}.$$

**Figure 1:**
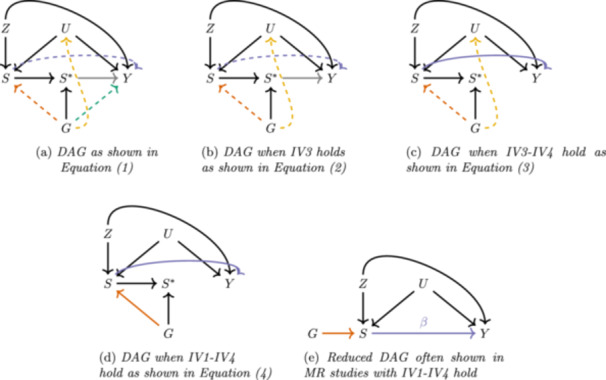
Causal diagrams (a–e) illustrating key assumptions in the paper.

Figure 1a depicts the directed acyclic graph consistent with the model described in Equation (1) and highlights various key assumptions using coloured arrows. We first consider the traditional set of assumptions required to estimate the ACE of the exposure on the outcome. We can express the ACE as the expected contrast between the potential outcomes of all mothers if they smoked during pregnancy, Y(S=1), and if they did not, Y(S=0):

ACE=E[Y(S=1)−Y(S=0)].

These assumptions are (Hernán and Robins 2020):

IV1 (relevance) : The genetic instrument G predicts the exposure S (orange arrow);
IV2 (independence) : The genetic instrument G is independent of any confounders U (**no** yellow arrow);
IV3 (exclusion) : The genetic instrument G is independent of the outcome Y given the exposure S and any confounders U (**no** green arrow).
IV4 (homogeneity) : The effect of the exposure S on the outcome Y is independent of the genetic instrument G (**no** grey arrow).

Assumptions IV1–4 enable us to extract the ACE via an IV analysis, by turning the general model and causal diagram in Figure 1 into the reduced model and causal diagram in Figure 1, through the following steps:

$$\begin{array}{ccc} E[Y|S,G,Z,U] & = & \gamma_{0}+\beta_{0}S+(\beta_{1}-\beta_{0})S^{*}+\gamma_{YG}G+\gamma_{YZ}Z+\gamma_{YU}U \end{array},$$

(2) $$\begin{array}{ccc} \text{(from}\;\text{IV3)} & = & \gamma_{0}+\beta_{0}S+(\beta_{1}-\beta_{0})S^{*}+\gamma_{YZ}Z+\gamma_{YU}U \end{array},$$

(3) $$\;\text{(from IV4)}\;=\gamma_{0}+\beta S+\gamma_{YZ}Z+\gamma_{YU}U,$$

(4) $$\;\text{(from IV1 and IV2)}\;=\gamma_{0}+\beta \hat{S}+\gamma_{YZ}Z+\epsilon_{Y},$$

where $\hat{S}=E\left[S|G\right]$ and $\epsilon_{Y}$ is a residual error term that is crucially independent of $\hat{S}$. The reduced causal diagram in Figure 1e is often shown in MR studies.

### 2.1 What Does an MR Analysis Estimate Under Violation of IV2–4?

We now consider what is targeted by the Wald ratio estimate for the causal effect in an MR analysis, assuming the data model described in Equation (1), when IV1 holds but initially, assumptions IV2–V4 do not. Note that we do not include the measured confounder *Z* in the following derivations as any bias through *Z* can be adjusted for. Under our assumed model as described in Equation (1), the Wald ratio estimate can be expressed as:

(5) $$\begin{array}{c} \frac{Cov\left(G,Y\right)}{Cov\left(G,S\right)}=\frac{\beta_{1}E\left[S|G=1\right]-\beta_{0}E\left[S|G=0\right]}{E\left[S|G=1\right]-E\left[S|G=0\right]}+\frac{\gamma_{YG}+\gamma_{YU}\left(E\left[U|G=1\right]-E\left[U|G=0\right]\right)}{E\left[S|G=1\right]-E\left[S|G=0\right]}=\frac{\beta_{1}E\left[S|G=1\right]-\beta_{0}E\left[S|G=0\right]}{E\left[S|G=1\right]-E\left[S|G=0\right]}+\;B\;\text{.} \end{array}$$

IV1 guarantees that the denominator of Equation (5) is nonzero and so the ratio terms are well defined. If homogeneity is violated, but monotonicity holds, we show in Supporting Information that Equation (5) equals the Complier Average Causal Effect (CACE) plus any bias due to violation of IV2 and IV3 (B term). Compliers are defined as individuals that smoke if they have the risk allele (G=1) and do not smoke if they do not have it (G=0).

### 2.2 Genetically Moderated Exposure Effect (GMEE)

Genetic instruments that satisfy the homogeneity assumption enable estimation of the ACE. However, in studies into the consequences of smoking versus not smoking, this assumption will be demonstrably false if attempting the analysis with a SNP like rs1051730, since the smoking patterns of people with and without this variant are likely to be different. In this case, a more practical starting point would be to assume the underlying DAG structure in Figure 1a and aim to quantify the magnitude of homogeneity violation as the difference in smoking effects between the two genetic sub‐groups. This ‘genetically moderated exposure effect’ (GMEE) is represented by arrow between $S^{*}$ and the outcome Y. From Equation (1) this is equal to $\beta_{1}-\beta_{0}$.

Bowden et al. (2021) discuss various methods for estimating this quantity, which we refer to as the GMEE, but which they referred to as the GMTE (T being for treatment). Each of the methods presented in Bowden et al. (2021) relies on a different set of assumptions. For example, when the genetic instrument G is independent of the exposure (i.e., no orange arrow in Figure 1 due to violation of IV1), is independent of any unmeasured confounder (i.e., no yellow arrow in Figure 1 and IV2 satisfied), and only affects the outcome through the moderated exposure variable (i.e., no green arrow in Figure 1 and IV3 satisfied), the GMEE can be estimated in the exposed population only. In our setting, this would be estimated by the difference in mean outcomes across the genetic groups among the population of smokers only:

$$\mathrm{GMEE}\left(1\right)=\hat{E}\left[Y|S=1,G=1\right]-\hat{E}\left[Y|S=1,G=0\right].$$

Here the ‘(1)’ notation reminds the analyst that only smoker's data is used and G=1∕0 refers to the presence/absence of at least one risk allele of SNP rs1051730. A more robust estimate of the GMEE is the

RGMEE=GMEE(1)−GMEE(0),

where

$$\mathrm{GMEE}\left(0\right)=\hat{E}\left[Y|S=0,G=1\right]-\hat{E}\left[Y|S=0,G=0\right].$$

Here, the *R* prefix in RGMEE stands for ‘robust’, since it can estimate the GMEE without bias even if IV3 is violated (i.e., G affects the outcome directly as indicated by the green arrow in Figure 1). Indeed it is this bias term that is estimated by GMEE(0) before being subtracted out.

Bowden et al. (2021) state that the RGMEE is unbiased even if the genetic instrument violates IV2, by being associated with the outcome through the unmeasured confounder (yellow arrow in Figure 1). Our investigations in this paper have shown this to be incorrect (see Supporting Information: Section 6). Nevertheless, this actually makes it more straightforward to verify if the assumptions for the RGEE hold (i.e., a desired violation of IV1 but no violation of IV2), since they imply that G and S are independent. Testing for an association between G and S is therefore an important prerequisite for its use.

## 3 Enhancing Robustness Through the Integration of MR and GMEE Methods

**Figure 2:**
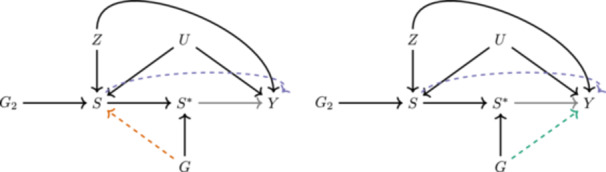
Left: DAG of linear interaction model from Equation (1) highlighting in colour which assumptions need to hold for a consistent (i.e., asymptotically unbiased) estimate of $\beta_{1}$ and $\beta_{0}$ when using Method 1. Right: DAG of linear interaction model (1) highlighting in colour which assumptions need to hold for a consistent estimate of $\beta_{1}$ and $\beta_{0}$ when using Method 2.

The genetically moderated exposure effect introduced in the previous section proposes an array of methods for estimating the difference $\beta_{1}-\beta_{0}$ under different assumptions, but not the individual values $\beta_{1}$ and $\beta_{0}$. To address this, we now formally extend the previous framework by incorporating a second variant, $G_{2}$, that is a ‘standard’ instrument for the exposure satisfying assumptions IV1–4. In our case, it therefore influences smoking initiation directly, but does not moderate an individual's smoking habits, thereby violating homogeneity. We now explore two scenarios that expand upon the standard TWIST approach, utilising novel methods that leverage the two available genetic instruments. The DAGs for these two separate methods are shown in Figure 2.

### 3.1 Method 1: $\left(G,G_{2}\right)$ are Jointly Valid Instruments for ($S,S^{*}$)

We first consider estimation of $\beta_{1}$ and $\beta_{0}$ using genetic instruments G and $G_{2}$ within a multivariable model. This can be enacted in a two‐step procedure by using G and $G_{2}$ to predict S in stage 1, and then plugging in the predicted values into the linear interaction model in stage 2:

1. Stage 1: Estimate $\hat{S}$ with a consistent estimate of $\hat{E}\left[S|G,G_{2}\right]$;
2. Stage 2: $Y=\gamma_{0}+\beta_{1}\hat{S}G+\beta_{0}\hat{S}\left(1-G\right)$, with $\hat{S}$ being the fitted values from stage 1.

This approach is robust to the case where the (assumed) effect modifying variant G violates IV1 but satisfies IV2 and IV3 (Figure 2, Left). In Supporting Information, we show through simulation that the true values of $\beta_{1}$ and $\beta_{0}$ can be recovered when these assumptions are satisfied, but violation of the assumptions lead to bias. The standard error for $\beta_{1}$ and $\beta_{0}$ can be obtained directly from the linear model output. As both parameters are estimated in the same model we can use the covariance matrix of $\beta_{1}$ and $\beta_{0}$ to derive the variance of $\beta_{1}-\beta_{0}$ and hence can estimate the standard error for $\beta_{1}-\beta_{0}$.

### 3.2 Method 2: Allowing for a Pleiotropic Effect of G on Y

We now propose a robust procedure that combines the general MR approach with the RGMEE given in Bowden et al. (2021). We first apply the RGMEE method to consistently estimate the genetically moderated effect $\beta_{1}-\beta_{0}$. We then define a new variable $Y\left(S^{*}=0\right)$ created by subtracting the genetically moderated effect times the moderated exposure from the original outcome Y. More formally, $Y\left(S^{*}=0\right)$ is a potential outcome in which the treatment effect of $S^{*}$ on Y has been set to zero. It is equal to Y (and therefore observed) for individuals with an $S^{*}$ = 0, but is unobserved for those with $S^{*}$ = 1. Finally, we perform an MR analysis using the genetic instrument $G_{2}$, the exposure S and $Y\left(S^{*}=0\right)$. This enables estimation of $\beta_{0}$, which can then be used in combination with the RMGEE to estimate $\beta_{1}$:

1. Estimate the RGMEE $\hat{\left(\beta_{1}-\beta_{0}\right)}$ using G;
2. Estimate $\hat{S}$ with a consistent estimate of $\hat{E}\left[S|G_{2}\right]$;
3. Estimate $\beta_{0}$ from model $E\left[Y\left(S^{*}=0\right)|\hat{S}\right]=\gamma_{0}+\beta_{0}\hat{S}$, where $Y\left(S^{*}=0\right)=Y-\hat{\left(\beta_{1}-\beta_{0}\right)}S^{*}$.

Method 2 delivers consistent estimates if the RGMEE estimate can be consistently estimated using G and $\beta_{0}$ can be consistently determined using $G_{2}$ once the GMEE effect has been removed. Compared to Method 1, it allows a direct pleioptropic effect of G on Y (IV3 violation) but requires G to be independent of S (IV1 violated, but IV2 satisfied). When these assumptions are not met, our simulations show that it leads to bias (see Supporting Information). The standard error for $\beta_{0}$ and $\beta_{1}-\beta_{0}$ can be directly taken from the respective model output. We make the assumption that $\hat{\beta_{1}-\beta_{0}}$ is independent of ${\hat{\beta }}_{0}$, so that S.E($\beta_{1}$) $\approx \sqrt{Var\left(\hat{\beta_{1}-\beta_{0}}\right)+Var\left({\hat{\beta }}_{0}\right)}$. In simulations we show that it leads to confidence intervals (CI) with only a slightly conservative coverage.

### 3.3 What Does the Standard MR Estimate Using $G_{2}$ as the IV Target?

When including a homogeneity respecting instrument as shown in Figure 2, a standard MR analysis with $G_{2}$ as the IV is possible. Using the two‐stage regression approach means:

1. Stage 1: Estimate $\hat{S}$ with a consistent estimate of $\hat{E}\left[S|G_{2}\right]$;
2. Stage 2: $Y=\alpha_{0}+\alpha_{1}\hat{S}+\alpha_{2}Z+\epsilon_{Y}$.

Here, $\alpha_{1}$ is the ACE on the outcome Y if all mothers where exposed compared to if all mothers were not exposed: E[Y(S=1)]−E[Y(S=0)]. It can be shown that under the model described in Figure 2 and Equation (1):

(6) $$\alpha_{1}=\beta_{0}+\left(\beta_{1}-\beta_{0}\right)E\left[G|S=1\right].$$

## 4 Simulation Results

### 4.1 Data Generation

We simulated data consistent with Figure 2 in the following manner:

(7) $$\begin{array}{ccc} G & \sim & ℬ\left(0.55\right),\\ G_{2} & \sim & ℬ\left(0.4\right),\\ U & = & \gamma_{UG}G+N\left(0,1\right),\\ \eta & = & -2+\gamma_{SG}G+\gamma_{SG_{2}}G_{2}+\gamma_{SU}U+N\left(0,0.5\right),\\ p_{S} & = & \frac{\text{exp}\left(\eta \right)}{1+\text{exp}\left(\eta \right)},S\sim ℬ\left(p_{S}\right),\\ Y & = & 3500+\beta_{1}SG+\beta_{0}S\left(1-G\right)+\gamma_{YG}G+\gamma_{YU}U+N\left(0,470\right). \end{array}$$

The outcome (Y) model in (7) was chosen so that simulated data closely matched real birth weight data (in grams) for mothers with a history of smoking in the Avon Longitudinal Study of Parents and Children (ALSPAC) (Boyd et al. 2013; Fraser et al. 2013; Northstone et al. 2023) which we will subsequently use in our applied analysis. By choosing zero and nonzero values for the parameters $\gamma_{UG},\gamma_{SG}$ and $\gamma_{YG}$, we were able to explore the performance of Methods 1 and 2 in estimating the causal effect parameters $\beta_{1}$ and $\beta_{0}$. We choose to set $\beta_{1}=-200$ and $\beta_{0}=-100$, which assumes a genetically moderated effect of $\beta_{1}-\beta_{0}=-100$ g. For all simulations, we made sure that the assumptions of Methods 1 and 2 held. Each simulation was repeated N=20,000 times, which enabled the calculation of bias, coverage and statistical power. For further details, Supporting Information Table S2 provides a summary of the simulated data under all of the explored scenarios.

### 4.2 Estimation Accuracy With Increasing Sample Size

**Figure 3:**
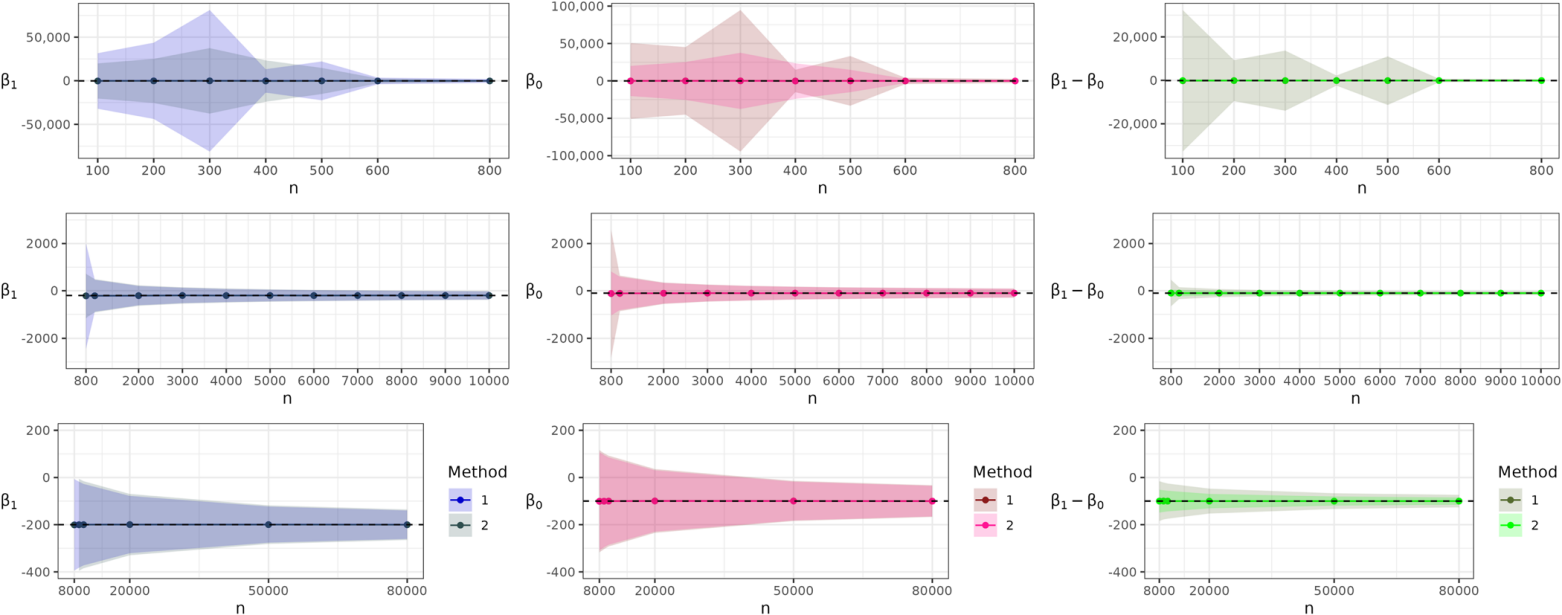
Mean estimates for $\beta_{1},\beta_{0}$ and $\beta_{1}-\beta_{0}$ over 20,000 simulations for different sample sizes. The shaded area shows the 95% CI interval derived with the Monte Carlo standard error. The dashed line indicates the true value.

We investigated the sample size needed to unbiasedly estimate $\beta_{1}$ and $\beta_{0}$ using each approach when their respective assumptions were satisfied. Data sets were generated with sample sizes between 100 and 80,000 individuals. Figure 3 shows the mean values of $\beta_{1},\beta_{0}$ and $\beta_{1}-\beta_{0}$ using Methods 1 and 2 across 20,000 simulations. Shaded areas reflect, for each mean parameter estimate obtained from a given sample size, a 95% CI calculated as ±1.96×SD(.) around the mean, SD(.) being the standard deviation of the 20,000 estimates (Morris et al. 2019). Note that for each estimate we display three subfigures (per column) with a different range on the *y*‐axis: one for small sample sizes, one for medium size sample sizes and one for large sample sizes. Details on the parameter values used for each simulation are described in Supporting Information. Estimation of $\beta_{1}$ and $\beta_{0}$ become more precise with shrinking CIs as the sample size increases. Both, Methods 1 and 2 lead to similar results. However, for small sample sizes (below 2000), CIs for Method 1 estimates are wider. The third column of Figure 3 shows the mean estimates for $\beta_{1}-\beta_{0}$ using Methods 1 and 2. Here we can see a distinction in the performance of the methods across all sample sizes. Method 2, which uses the RGMEE method, yields narrower CIs for $\hat{\beta_{1}-\beta_{0}}$ even for small sample sizes.

### 4.3 Power and the coverage

**Figure 4:**
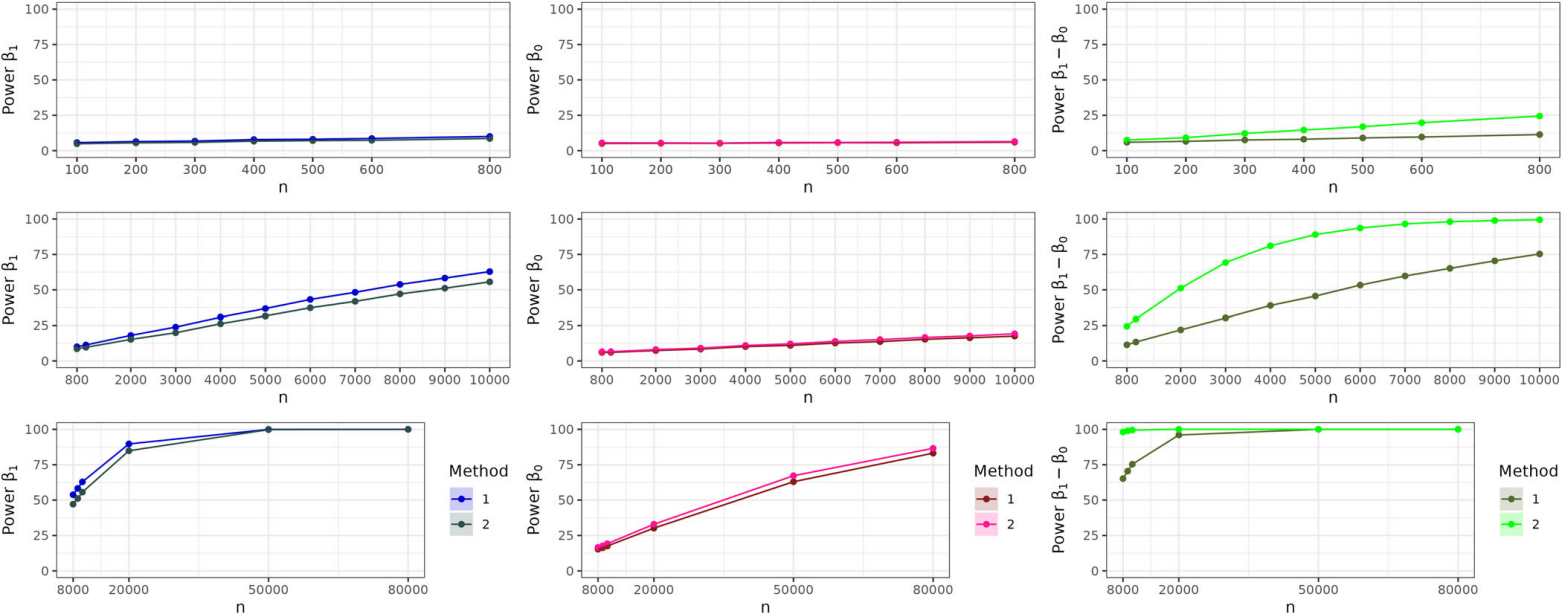
Power for $\beta_{1},\beta_{0}$ and $\beta_{1}-\beta_{0}$ over 20,000 simulations for different sample sizes. The shaded area shows the 95% CI interval for the power derived with the Monte Carlo standard error.

**Figure 5:**
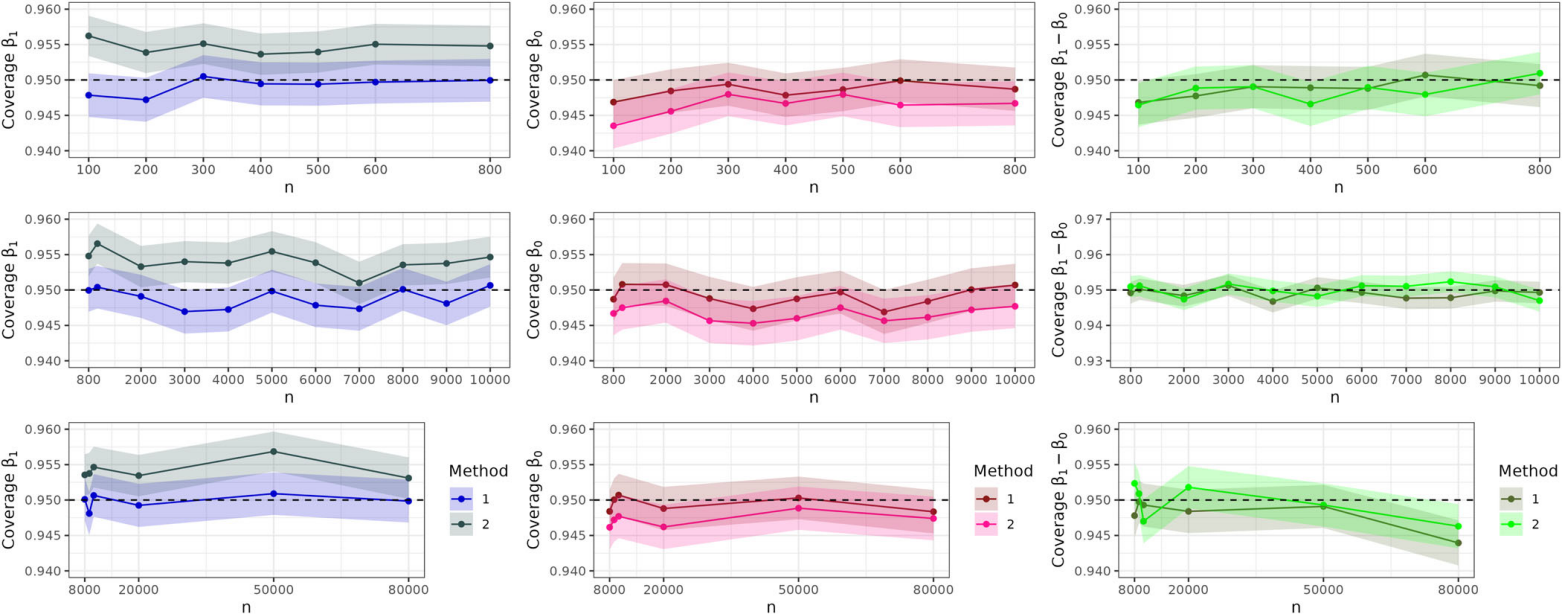
Coverage for $\beta_{1},\beta_{0}$ and $\beta_{1}-\beta_{0}$ over 20,000 simulations for different sample sizes. The shaded area shows the 95% CI interval for the coverage derived with the Monte Carlo standard error.

We estimated the power to reject the null hypothesis that $\beta_{1},\beta_{0}$ and $\beta_{1}-\beta_{0}$ were statistically different from zero at the 5% significance level, using Methods 1 and 2. For each simulation, we also calculated CIs for the parameter estimates based on estimated standard errors, and reported the coverage of 95% CIs across the 20,000 simulations. The results for power and coverage are shown in Figures 4 and 5 respectively, along with their Monte‐Carlo standard errors (Morris et al. 2019). Our results show that Method 2 results in a higher power when estimating $\beta_{0}$ and $\beta_{1}-\beta_{0}$ than Method 1 for a given sample size. However, when considering estimation of $\beta_{1}$, this is reversed. The power to detect $\beta_{0}$ is lower than the power to detect $\beta_{1}$ due to its lower effect size of −100 (compared to $\beta_{1}=-200$). Figure 5 reveals a near nominal coverage for both methods close to 95%. Crucially, our assumption that $\hat{\beta_{1}-\beta_{0}}$ and ${\hat{\beta }}_{0}$ are independent leads only to a slightly conservative coverage when estimating a CIs for $\beta_{1}$ with Method 2.

## 5 Applied Example

### 5.1 Biological Example for Genetically Driven Exposure Effects

Research into the adverse consequences of smoking has been ongoing since the 1950s, up until the present day (Doll and Hill 1950; U.S. Department of Health and Human Services 2014). In the specific context of maternal health, it is well established that smoking during pregnancy is associated with lower offspring birth weight, which is itself an important predictor of infant mortality and many later life health outcomes, such as cardiovascular disease, high blood pressure, coronary heart disease and type 2 diabetes (Moen et al. 2020; Tyrrell et al. 2012; Warrington et al. 2019). Attributing the correct proportion of these estimated associations that are due to the causal consequences of smoking is not straightforward, due to strong confounding between smoking and later life outcomes by socioeconomic factors which are very hard to completely control for. Despite this, smoking is viewed as a key modifiable risk factor, and reducing its prevalence during pregnancy remains an important public health target (Cnattingius 2004; U.S. Department of Health and Human Services 2014). Unfortunately, NHS digital service statistics indicate that approximately 8.6% of UK mothers were known smokers at the time of delivery in the first half of 2023 (Population Health, Clinical Audit, Team, Specialist Care, & Lead Analyst: Walt Treloar 2023). Identifying which individuals are at a higher risk of not giving up smoking and therefore might face more severe pregnancy outcomes can be crucial when targeting smoking cessation programmes, to provide support as well as closer monitoring during pregnancy.

Recently, genome‐wide association studies (GWAS) have identified genetic variants that are associated with smoking initiation, smoking cessation, the age of starting smoking and smoking quantity (Liu et al. 2019). Freathy et al. (2009) show that rs1051730 on chromosome 15 is associated with smoking cessation during pregnancy as well as smoking quantity. A strong biological rationale for this exists as rs1051730 is in the nicotine acetylcholine receptor gene cluster *CHRNA5‐CHRNA3‐CHRNB4*. Rare variant burden associations have implicated all three of these genes as important in influencing smoking quantity (Rajagopal et al. 2023). However, it has also been shown that rs1051730 is *not* associated with smoking initiation (Freathy et al. 2009). The methods we have introduced thus far appear well suited to estimating the causal effect of smoking on birth weight using traditional genetic instruments for smoking initiation, whilst at the same time, quantifying the genetically moderated smoking effect via rs1051730.

### 5.2 The Effect of Smoking on Birth Weight in the ALSPAC Study

The Avon Longitudinal Study of Parents and Children (ALSPAC) (Boyd et al. 2013; Fraser et al. 2013; Northstone et al. 2023) invited pregnant women resident in Avon, UK with expected dates of delivery between 1 April 1991 and 31 December 1992, to take part in the study. The initial number of pregnancies enrolled was 14,541. Of the initial pregnancies, there was a total of 14,676 foetuses, resulting in 14,062 live births and 13,988 children who were alive at 1 year. We restricted our analysis to unrelated mothers with available genetic information. Additionally, we excluded multiple births and preterm births (pregnancy duration ≤37 weeks) (Jaitner et al. 2024). The analysis data set had a sample size of 7752 individual mothers. For the traditional genetic instrument ‘$G_{2}$’ we created a weighted genetic risk score (GRS) among the smoking initiation SNPs identified by the latest GWAS (Liu et al. 2019). The effect sizes from the same GWAS were used as weights. We used rs1051730 as genetic effect‐modifying instrument ‘G’, coded as 0 and 1 corresponding to no and at least one risk allele respectively. Various different smoking definitions were used for the exposure outlined in the following sections. The ALSPAC study website contains details of all the data that are available through a fully searchable data dictionary and variable search tool (http://www.bristol.ac.uk/alspac/researchers/our-data/).

#### 5.2.1 Exposure S is Smoking Before Pregnancy

**Figure 6:**
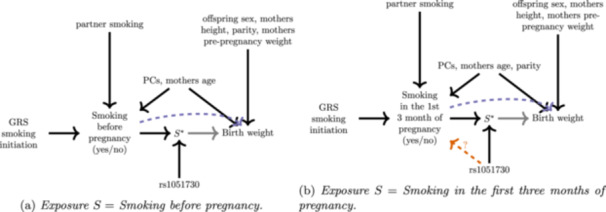
DAG shows the relationships between the genetic instrument, the exposure, the outcome and how those are affected by different confounders. $S^{*}=G$ x S represents the interaction between the genetic variant rs1051730 and the exposure S.

**Figure 7:**
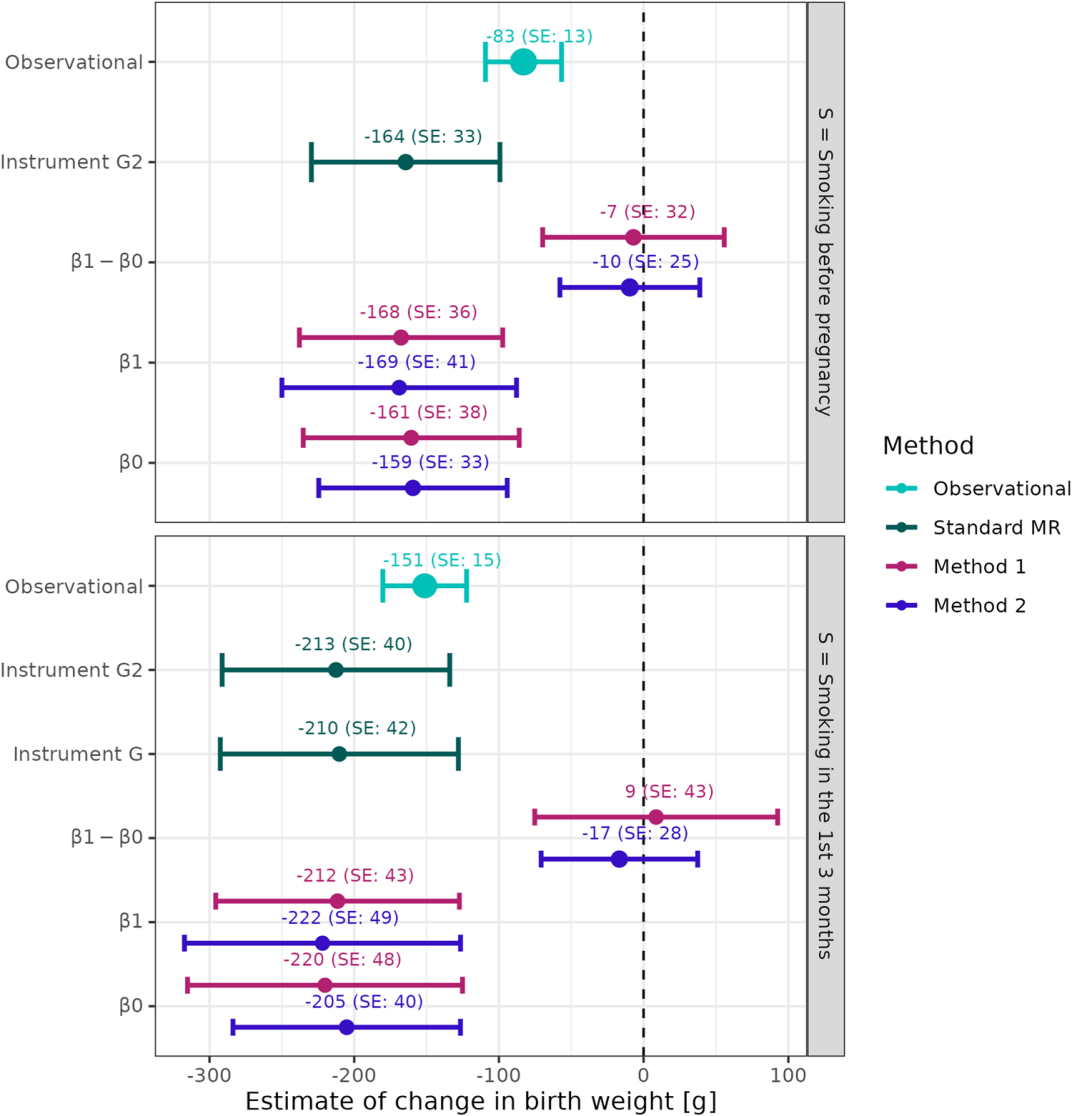
$\beta_{1}-\beta_{0},\beta_{1}$ and $\beta_{0}$ estimates from applying Methods 1 and 2 to the ALSPAC data set. Bars indicate the 95% confidence interval. Observational analysis results arise from a linear regression of the smoking variable on birth weight adjusted for partner smoking, mothers' age, mothers' height, mothers' pre‐pregnancy weight, parity, and offspring sex. Dark green are the results from the individual level data MR analysis using the two‐stage least squares method using G or $G_{2}$ as the instrument, S as the exposure and birth weight is the outcome. G has no association with S= smoking before pregnancy, hence we did not perform an MR analysis. The results for applying Methods 1 and 2 to estimate $\beta_{1}-\beta_{0},\beta_{1}$ and $\beta_{0}$ are shown in red and blue respectively.

Each mother was asked at 16–18 weeks of gestation whether she smoked before pregnancy. We coded mothers that reported ‘yes’ as S=1 and mothers who reported ‘no’ as S=0. Figure 6a displays the assumed DAG for our analysis. We aimed to apply Methods 1 and 2 to estimate the causal effect of pre‐pregnancy smoking on birth weight in the G=1 group, $\beta_{1}$, the G=0 group, $\beta_{0}$, and also the genetically moderated exposure effect $\beta_{1}-\beta_{0}$. We would expect this latter quantity to be nonzero if the pre‐pregnancy smoking effect persisted differently throughout pregnancy across the two genetic groups. For the first stage of Method 1, we perform a logistic regression of S on the GRS of smoking initiation ($G_{2}$) and rs1051730 (G). The results are shown in Table 1. Variant rs1051730 was not associated with smoking before pregnancy, which helpfully means that Method 2 is not ruled out as an analysis option. The GRS is also associated with smoking before pregnancy and we assume it acts as a true IV for this exposure. Two crucial assumptions are that the GRS of smoking initiation has no pleiotropic effect on birth weight and it does not modify the the causal effect between smoking and birth weight in the exposed and the unexposed. To apply Method 1, rs1051730 cannot have a pleiotropic effect on birth weight either but, for Method 2, this assumption is relaxed. The results from applying both methods are shown in Figure 7. To increase the precision of the estimates we adjust our regression models for different sets of covariates. The model for the genetic prediction of smoking is adjusted for whether the partner of the mother smoked, the mothers' age and the first 10 genetic principal components. The model predicting birth weight is adjusted for offspring sex, mother age, mothers' height, parity, mothers' prepregnancy weight and the first 10 genetic principal components. We viewed these variables as confounders for either smoking before pregnancy or birth weight or both. For mothers who have at least one G risk allele, the ACE of smoking before pregnancy, $\beta_{1}$, was estimated to be a 168 and 169 g reduction in birth weight using Methods 1 and 2 respectively. On the other hand, the corresponding causal effect ($\beta_{0}$) in smoking mothers without a rs1051730 risk allele is a 159 and 161 g birth weight reduction for Methods 1 and 2 respectively compared to nonsmoking mothers without the risk allele.

**Table 1:** Logistic regression model results for $\mathrm{logit}\left(Pr\left(S=1\right)\right)=\alpha_{0}+\alpha_{1}G+\alpha_{2}G_{2}+\delta^{T}Z$ as the first stage of Method 1 using pre‐pregnancy smoking as the outcome variable $S.\;G_{2}$ = smoking initiation GRS, G = rs1051730 and Z is a vector of covariates.

S =		Est.	Std. Error	*p*‐value	*F* statistic
Smoking before pregnancy	rs1051730 (G)	${\hat{\alpha }}_{1}$ = 0.02	0.06	0.73	0.12
	GRS smoking initiation ($G_{2}$)	${\hat{\alpha }}_{2}$ = 1.46	0.16	4.85e‐20	84
Smoking in the first 3 months	rs1051730 (G)	${\hat{\alpha }}_{1}$ = 0.13	0.07	0.06	3.55
	GRS smoking initiation ($G_{2}$)	${\hat{\alpha }}_{2}$ = 1.39	0.18	2.58e‐15	62.6

#### 5.2.2 Exposure S is Smoking in the First 3 Months of Pregnancy

Mothers were asked at 16–18 weeks of gestation whether they smoked in the first 3 months of pregnancy. We coded those that reported ‘yes’ as S=1 and mothers who reported ‘no’ as S=0 and proceeded to estimate the causal effect of this exposure at the two levels (no and at least one risk allele) of rs1051730 and the genetically moderated exposure effect. In this analysis, no information about smoking before pregnancy was taken into account and therefore the mothers reporting to be non‐smokers either gave up smoking when getting pregnant or did not smoke before pregnancy. Figure 6b displays the assumed DAG for our analysis. Methods 1 and 2 were applied to derive estimates for $\beta_{1},\beta_{0}$ and $\beta_{1}-\beta_{0}$, with results shown in Table 1 and Figure 7. For these analyses, the logistic model for S given G and $G_{2}$ was adjusted for whether the partner of the mother smokes, the mothers' age, parity and the first 10 genetic principal components. The model predicting birth weight was adjusted for offspring sex, mothers' age, mothers' height, parity, mothers' prepregnancy weight and the first 10 genetic principal components. We viewed these variables as confounders for either smoking before pregnancy or birth weight or both. For this analysis, a stronger association was observed between rs1051730 and S, meaning that we were cautious with the interpretation of Method 2 results, given its crucial role in the in estimation of the genetically moderated exposure effect. The causal effect of smoking during pregnancy on birth weight in those with a rs1051730 risk allele, $\beta_{1}$, was estimated to be −212 and −222 g using Methods 1 and 2 respectively, whereas the effect of smoking in the individuals without the genetic variant on birth weight is −220 and −205 g for Method 1 and 2 respectively.

For both smoking exposures, we were unable to identify a difference between the two genetic groups. This could be because the effect of pre‐pregnancy smoking and smoking in the first 3 months does not truly differ in people with or without variant rs1051730. However, a simulation investigation showed that large numbers of individuals would be required to identify a genetically driven effect of smoking with the magnitudes we observed in our analysis. Specifically, we simulated data with sample sizes from 7000 to 500,000 and a true difference between the genetic groups of $\beta_{1}-\beta_{0}$ between −20 and −5 g. To reach 80% power in estimating $\beta_{1}-\beta_{0}=-15$ a sample size of 500,000 individuals is required in our simulation when using Method 1. The RGMEE (Method 2), which is more robust to pleiotropy compared to Method 1, is able to estimate $\beta_{1}-\beta_{0}=-15$ with a power of over 80% with 200,000 individuals. More details on the results of this are shown in Supporting Information: Section 5. This provides important guidance on the much larger sample size, way beyond the 7752 mothers in the ALSPAC study, that would be required to detect a difference between the genetic groups in the region of what we observe here.

Despite not being able to detect a statistically meaningful genetically moderated exposure effect, overall our results suggest that smoking before pregnancy or smoking in the first 3 months of pregnancy results in a lower birth weight compared to not smoking. This is in line with previous publications (Larsen et al. 2018; Tyrrell et al. 012; Pereira et al. 2022).

### 5.3 Observational Analysis and ‘Standard’ MR

In addition to applying the new proposed methods to the ALSPAC data set, we also looked at the observational association between smoking and birth weight. A linear regression of S (using both smoking definitions) on birth weight adjusted for partner smoking, mothers' age, mothers' height, mothers' pre‐pregnancy weight, parity and offspring sex yielded negative associational estimates. Although the observational analysis likely suffers from residual confounding, and cannot be interpreted as a causal effect, the direction of effect remains the same compared to estimating $\beta_{1}$ and $\beta_{0}$ (Figure 7), albeit of a smaller magnitude.

As indicated in Table 1, rs1051730 (G) is not associated with smoking before pregnancy. Therefore, we are unable to perform a standard MR analysis using G as the genetic instrument for S= smoking before pregnancy. However, we did perform a standard MR analysis using individual level data and the two‐stage least squares approach with S= smoking in the first 3 months of pregnancy. We explain in Section 2.1 that the standard MR with a homogeneity violating instrument like rs1051730 estimates the CACE (while the monotonicity assumption holds). The CACE of smoking in the first 3 months of pregnancy on birth weight is −210 g (95% CI: [−293,−128]). Note that these results potentially suffer from weak instrument bias.

Additionally, and as a confirmatory test of our previous genetic analyses, we calculated a standard MR estimate using the GRS for smoking initiation to instrument smoking before and smoking in the first 3 months of pregnancy. For these analyses rs1051730 is not considered. The methodology is described in Section 3.3. Using Equation (6), the estimated β‐values obtained from applying Method 1 and 2 to the ALSPAC data we derive an ACE of −165 g for smoking before pregnancy. This compares to the ACE of −164 g (Figure 7) estimated with standard MR approach. Similarly, for smoking in the first 3 months of pregnancy we obtain an estimate of −213 g using the β‐values from Methods 1 or 2 outputs and the formula provided in Equation (6).

## 6 Discussion

In this study, we propose a general framework for MR that allows the inclusion of traditional genetic instruments, as well as those that violate the key homogeneity assumption. This enables an analysis that goes beyond estimation of the ACE to consider estimation within specific genetic sub‐groups, with a view to quantifying genetically driven effect heterogeneity. Our approach builds on ideas from the pharmacogenetic TWIST framework proposed by Bowden et al. (2021) to a more mainstream epidemiological setting, as well as incorporating the technique of multivariable MR (Sanderson et al. 2019). Specifically, Method 1 offers a new approach to estimating the genetically moderated exposure effect, which could be triangulated with existing methods. Furthermore, Method 1 allows for a direct effect between the homogeneity‐violating instrument and the exposure. In the presence of unmeasured confounding, the methods proposed in the paper by Bowden et al. (2021) require the instrument to be independent of the exposure. To allow for a direct pleiotropic effect between the homogeneity‐violating instrument and the outcome we proposed Method 2. Simulation studies revealed the necessary sample sizes to detect an effect with sufficient power under Methods 1 and 2, considering plausible genetically moderated exposure effect sizes. To motivate the methods, we applied them to data from the ALSPAC cohort to investigate the effect of smoking before and in pregnancy on birth weight using a traditional GRS for smoking and rs1051730 as an effect modifier.

Our work could be further extended by considering the incorporation of additional methods to allow for the relaxation of further key assumptions. For example, allowing a genetic variant with a pleiotropic effect that acts through an unmeasured confounder (i.e., correlated pleiotropy). We assumed that the underlying data structure follows a linear interaction model. Future work could explore different data structures and nonlinear effects. For simplicity, and to naturally follow on from the approach proposed in Bowden et al. (2021), we assumed a binary effect modifying instrument through the dichotomisation of the genetic instrument rather than using the number of risk alleles. Future work could relax this assumption.

Despite not being able to show a difference between the two genetic groups in our applied example due to a limitation in sample size, our investigation clarifies how large future cohort study samples need to be to estimate effects of the magnitude we observed. We believe our framework is a useful methodological extension to investigate genetically driven heterogeneity. Our methods could, for example, be applied in other setting where larger sample sizes are available, or by meta‐analysing results with additional cohorts. Settings outside of pregnancy research are also possible and would not require mother and child pair information. For example, investigating the genetically driven effect of continuing smoking on lung cancer. Other examples could be using genetic variants associated with reducing alcohol consumption and the effects on various health outcomes. Data sets like the UK Biobank with genetic information available for 500,000 individuals could be used. R code for implementing the methods can be found at https://github.com/AJaitner/paper_heterogeneity_MR as well as code to implement the simulation studies and applied analysis.

## Ethics Statement

Ethical approval for the study was obtained from the ALSPAC Ethics and Law Committee and the Local Research Ethics Committees. Informed consent for the use of data collected via questionnaires and clinics was obtained from participants following the recommendations of the ALSPAC Ethics and Law Committee at the time.

## Conflicts of Interest

Jack Bowden is a part‐time employee of Novo Nordisk, however, this work is unrelated to his role at the company. The remaining authors declare no conflicts of interest.

## Supporting information

Supplementary Information

## Data Availability

The data in ALSPAC is fully available, via managed systems, to any researchers. The managed system is a requirement of the study funders, but access is not restricted on the basis of overlap with other applications to use the data or on the basis of peer review of the proposed science. The ALSPAC data management plan describes in detail the policy regarding data sharing, which is through a system of managed open access. The following steps highlight how to apply for access to the data included in this paper and all other ALSPAC data. (1) Please read the ALSPAC access policy, which describes the process of accessing the data and samples in detail and outlines the costs associated with doing so. (2) You may also find it useful to browse the fully searchable ALSPAC research proposals database, which lists all research projects that have been approved since April 2011. (3) Please submit your research proposal for consideration by the ALSPAC Executive Committee. You will receive a response within 10 working days to advise you whether your proposal has been approved. If you have any questions about accessing data, please email alspac-data@bristol.ac.uk.
