# Supplementary material for: Exploring and Accounting for Genetically Driven Effect Heterogeneity in Mendelian Randomization
Source: Genet Epidemiol. 2024 Sep 22;49(1):e22587. doi: 10.1002/gepi.22587 (PMC11656040; doi:10.1002/gepi.22587)
Supplement: Supplementary file 1 — Supplementary Information [file GEPI-49-0-s001.pdf]

Exploring and accounting for genetically driven effect heterogeneity in Mendelian Randomization Supplementary material

Annika Jaitner[1*], Krasimira Tsaneva-Atanasova[2,3], Rachel M. Freathy[1], Jack Bowden[1,4]
[1]Department of Clinical and Biomedical Sciences, Faculty of Health and Life Sciences, University of Exeter, Exeter, UK
[2]Department of Mathematics and Statistics, Faculty of Environment, Science and Economy, University of Exeter, Exeter, UK
[3]EPSRC Hub for Quantitative Modelling in Healthcare University of Exeter, Exeter, UK
[4]Novo Nordisk Genetics Centre of Excellence, Oxford, UK
*Correspondence: a.jaitner@exeter.ac.uk

1 Appendix: MR estimate equals the Complier Average Causal Effect if homogeneity is violated but monotonicity holds

To better understand the bias term of (5) from Section 2.1 in the main paper in the presence of IV4 violation, we first introduce the Principle Stratum framework described in Angrist, Imbens, and Rubin [1]. In our context, we imagine the existence of four compliance classes:

- *Compliers*: Individuals that smoke if they have the risk allele ($G = 1$) and do not smoke if they do not have it ($G = 0$).
- *Always Smokers*: Individuals that always smoke regardless of their genotype.
- *Never Smokers*: Individuals that never smoke regardless of their genotype.
- *Defiers*: Individuals that go against their genotype, this means they smoke if they do not have the risk allele and do not smoke if they do have the risk allele.

Formally this can be written as in Table S1 which relates compliance classes (and their proportion in the population) to the joint values of two potential smoking variables, $S(G = 1)$ and $S(G = 0)$ [2]:

	$S(G=1)$	$S(G=0)$	Proportion
Compliers (c)	1	0	π_c
Always Smokers (as)	1	1	π_{as}
Never Smokers (ns)	0	0	π_{ns}
Defiers (d)	0	1	π_d

Table S1: *Relating compliance classes (and their proportion in the population) to the joint values of two potential smoking variables.*

If we assume IV4 is violated but an alternative assumption, that there are no *Defiers* ($\pi_d = 0$, also termed 'monotonicity') holds, then from Table S1, we can equate:

$$E[S|G=1] = \pi_{as} + \pi_c$$
$$E[S|G=0] = \pi_{as}$$

Hence, equation (5) can be rewritten as:

$$\frac{\beta_1 E[S|G=1] - \beta_0 E[S|G=0]}{E[S|G=1] - E[S|G=0]} + \text{B}$$
$$= \frac{\beta_1(\pi_{as} + \pi_c) - \beta_0 \pi_{as}}{\pi_c} + B$$
$$= \beta_c + B,$$

with β_c the causal effect of smoking in compliers. As shown in Bowden et al. [2] β_1 can also be expressed as a weighted average of the causal effect of smoking in compliers (β_c) and always smokers (β_0):

$$\beta_1 = \frac{\beta_c \pi_c + \beta_0 \pi_{as}}{\pi_c + \pi_{as}}.$$

Therefore, if homogeneity is violated, but monotonicity holds, MR targets the average causal effect of smoking among *Compliers* (the complier average causal effect(CACE)) plus any bias due to violation of IV2 and IV3.

2 Appendix: Mean and standard derivation across simulated and ALSPAC data sets

In Table S2 the mean and standard derivation for each variable are shown for the ALSPAC data set (n = 7752) and the different simulation data sets. Depending on the definition of smoking the results are slightly different. Therefore, we show the mean and variance for ALSPAC defining S as smoking before pregnancy (yes/no) and for smoking in the first three month of pregnancy (yes/no). As described in the main body of the paper, we simulate data sets with different sample sizes and applied all methods to them. For each method we assume that the required assumptions for the respective method hold. This means that the data generating model is slightly different for each method and this also reflects in the mean and standard derivation for each variable. As an example we show the mean and variance for each variables for the different methods for a data set with 50,000 individuals. Note that each simulation is repeated 20,000 times and therefore for each method 20,000 data sets are generated. Hence, the mean value of the 20,000 mean values is displayed (similarly for the standard derivation).

Data set	$\bar{Y}$ ($\sigma(Y)$)	$\bar{S}$ ($\sigma(S)$)	$\bar{G}$ ($\sigma(G)$)
ALSPAC, S = pre-pergnnacy smoking	3474.21 (476.87)	0.31 (0.46)	0.55 (0.50)
ALSPAC, S = smoking in the 1st 3 month	3474.21 (476.8741)	0.23 (0.42)	0.55 (0.50)
Method 1 simulation n= 50,000	3459.17 (476.86)	0.26 (0.44)	0.55 (0.50)
Method 2 simulation n= 50,000	3506.07 (477.35)	0.24 (0.43)	0.55 (0.50)

Table S2: *Mean and standard derivation of the outcome, smoking variable and rs1051730 in ALSPAC (n = 7752) and in simulated data sets with 50,000 individuals.*

3 Appendix: Simulation parameter values for each method

In order for the assumptions for each method to be satisfied, the data generating model needs to be slightly different. Here we show the parameter values used for the data generation for each method.

Method	γ_{UG}	γ_{SG}	γ_{SG_2}	γ_{SU}	γ_{YG}	γ_{YU}	β_1	β_0
1	0	0.2	0.8	1.6	0	80	-200	-100
2	0	0	0.8	1.6	80	80	-200	-100

Table S3: *Simulation parameter values to generate simulated data for method 1 and method 2.*

4 Appendix: Simulations to show which assumptions need to hold for each method

We simulated data following the data generation process described in the main paper in section 4.1. We aimed to match the parameters of the data generating model as close to the ALSPAC data set as possible. We choose to set $\beta_1 = -200$ and $\beta_0 = -100$, which assumes a genetically moderated effect of $\beta_1 - \beta_0 = -100$ and a twice as large effect in the $G = 1$ group compared to the $G = 0$ group. We chose scenarios where some of the assumptions hold and others do not hold. For all simulations we choose a sample size of $n = 20,000$ and repeated each simulation 20,000 times.

4.1 Simulation for method 1

We investigated 9 different scenarios and show the density plots for the estimation of β_1 and β_0. We expect unbiased estimations for scenario 1-6 and verify that method 1 is robust to whether there is a direct effect between G and S. Scenario 7-8 are biased due to direct and/or indirect pleiotropy of the genetic instrument G. The density plots over the 20,000 simulation runs for each scenario are shown in Figure S1. As expected, strength of the instrument influences the precision of the estimation and therefore we see in Figure S1 that the density peak is higher for scenario 3 and scenario 6 compared to the other scenarios. It is also clearly visible that the scenarios with pleiotropy of the genetic instrument G (scenario 7-9) lead to biased results.

	Description	γ_{SG}	γ_{SG_2}	γ_{YG}	γ_{UG}	F-Stat G	F-Stat G_2
1	Direct effect between G and S	0.2	0.4	0	0	71	19
2	Direct effect between G and S	0.8	0.8	0	0	309	302
3	Direct effect between G and S	2	2	0	0	1765	1818
4	No direct effect between G and S	0	0.4	0	0	69	1
5	No direct effect between G and S	0	0.8	0	0	286	1
6	No direct effect between G and S	0	2	0	0	1850	1
7	Indirect pleiotropy	0.8	0.8	0	0.8	310	2013
8	Direct pleiotropy	0.8	0.8	80	0	309	302
9	Indirect and direct pleiotropy	0.8	0.8	80	0.8	310	2014

Table S4: *Parameter values for the difference scenarios to verify the assumptions for method 1. Increasing strength of the instrument G and G_2 for scenario 1-3 and increasing strength of the instrument for G for scenario 4-6. The confounder parameters are set to $\gamma_{YU} = 80$, $\gamma_{SU} = 1.6$.*

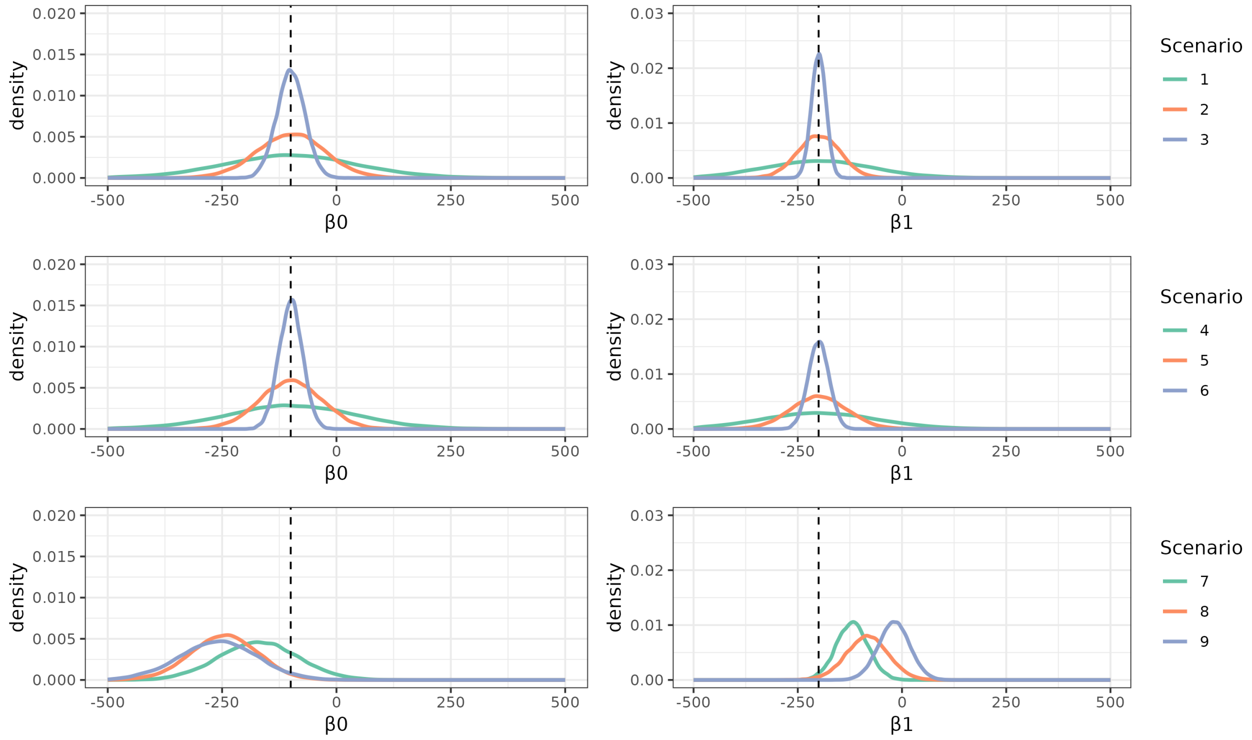

Figure S1: *Density plots for the estimation of β_0 and β_1 using method 1 over 20,000 simulation runs for 9 different scenarios.*

4.2 Simulation for method 2

We investigated 8 different scenarios and show the density plots for the estimation of β_0 and $\beta_1 - \beta_0$. We expect only scenario 1 and 2 to be unbiased. The direct effect between S and G bias the estimation of $\beta_1 - \beta_0$ and as this estimation is crucial for the following steps of estimating β_0. However, as the biased estimation is not a visible in Figure S2 we also provide the mean and standard derivation for the estimates for each scenario in Table S6. An effect between the genetic instrument G and the unmeasured confounder U and hence the indirect pleiotropy results in biased estimates for scenario 3-8. However, a direct pleiotropic effect of G is no problem (scenario 2 is unbiased)

	Description	γ_{SG}	γ_{SG_2}	γ_{YG}	γ_{UG}
1	No direct effect between G and S, no pleiotropy	0	0.8	0	0
2	No direct effect between G and S , direct pleiotropy	0	0.8	80	0
3	No direct effect between G and S, indirect pleiotropy	0	0.8	0	0.8
4	No direct effect between G and S , indirect and direct pleiotropy	0	0.8	80	0.8
5	Direct effect between G and S, no pleiotropy	0.8	0.8	0	0
6	Direct effect between G and S, direct pleiotropy	0.8	0.8	80	0
7	Direct effect between G and S, indirect pleiotropy	0.8	0.8	0	0.8
8	Direct effect between G and S, indirect and direct pleiotropy	0.8	0.8	80	0.8

Table S5: *Parameter values for the difference scenarios to verify the assumptions for method 2. The confounder parameters are set to $\gamma_{YU} = 80$, $\gamma_{SU} = 1.6$.*

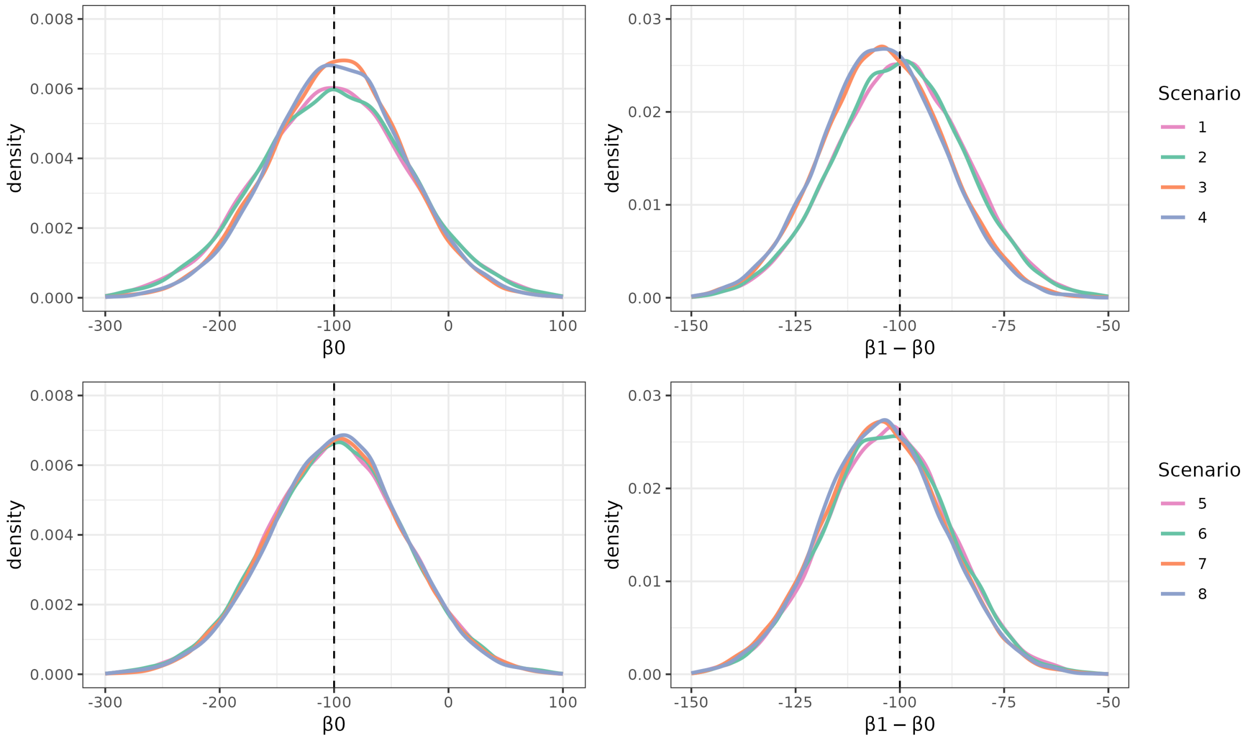

Figure S2: *Density plots for the estimation of β_0 and $\beta_1 - \beta_0$ using method 2 over 20,000 simulation runs for 8 different scenarios.*

	β_0 (95% CI)	$\beta_1 - \beta_0$ (95% CI)	F-Stat G_2
1	**-100.69** (-101.59, -99.78)	**-99.78** (-100.00,-99.56)	285
2	**-100.21** (-101.12, -99.30)	**-100.08** (-100.30, -99.86)	286
3	-98.01 (-98.80,-97.21)	-103.84 (-104.05, -103.64)	303
4	-97.22 (-98.02,-96.42)	-104.21 (-104.41, -104.00)	303
5	-98.08 (-98.90, -97.26)	-102.84 (-103.05, -102.63)	304
6	-98.33 (-99.15, -97.51)	-103.07 (-103.27, -102.86)	305
7	-97.64 (-98.45, -96.84)	-104.18 (-104.39, -103.98)	278
8	-97.47 (-98.28, -96.66)	-104.13 (-104.34, -103.93)	279

Table S6: *Mean values and 95% confidence intervals for the estimation of β_0 and $\beta_1 - \beta_0$ using method 2 for different scenarios. Estimates for scenario 1 and 2 are highlighted in bold because the true value (-100) lies within the 95 % confidence interval.*

5 Appendix: Simulation of power for small differences between genetic groups

To investigate at which sample size the two methods are able to estimate a small $\beta_1 - \beta_0$ with sufficient power, we simulated different data sets. The data generation follows the same procedure as described in the main body of this paper. The parameter values γ are kept the same as in previous simulations. We investigated 4 different scenarios as shown in Table S7. Each scenario was simulated with sample sizes varying from 7,000 to 500,000. Each simulation was repeated 5,000 times ($N = 5,000$). We estimate the power to reject the null hypothesis that $\beta_1 - \beta_0$ were statistically different from zero at the 5% significance level across the 5,000 simulations. The results for both methods are shown in Figure S3.

Scenario	β_1	β_0	$\beta_1 - \beta_0$
1	-220	-200	-20
2	-200	-185	-15
3	-180	-170	-10
4	-168	-163	-5

Table S7: *Four different scenarios with small values for $\beta_1 - \beta_0$, for data simulations with different large sample sizes.*

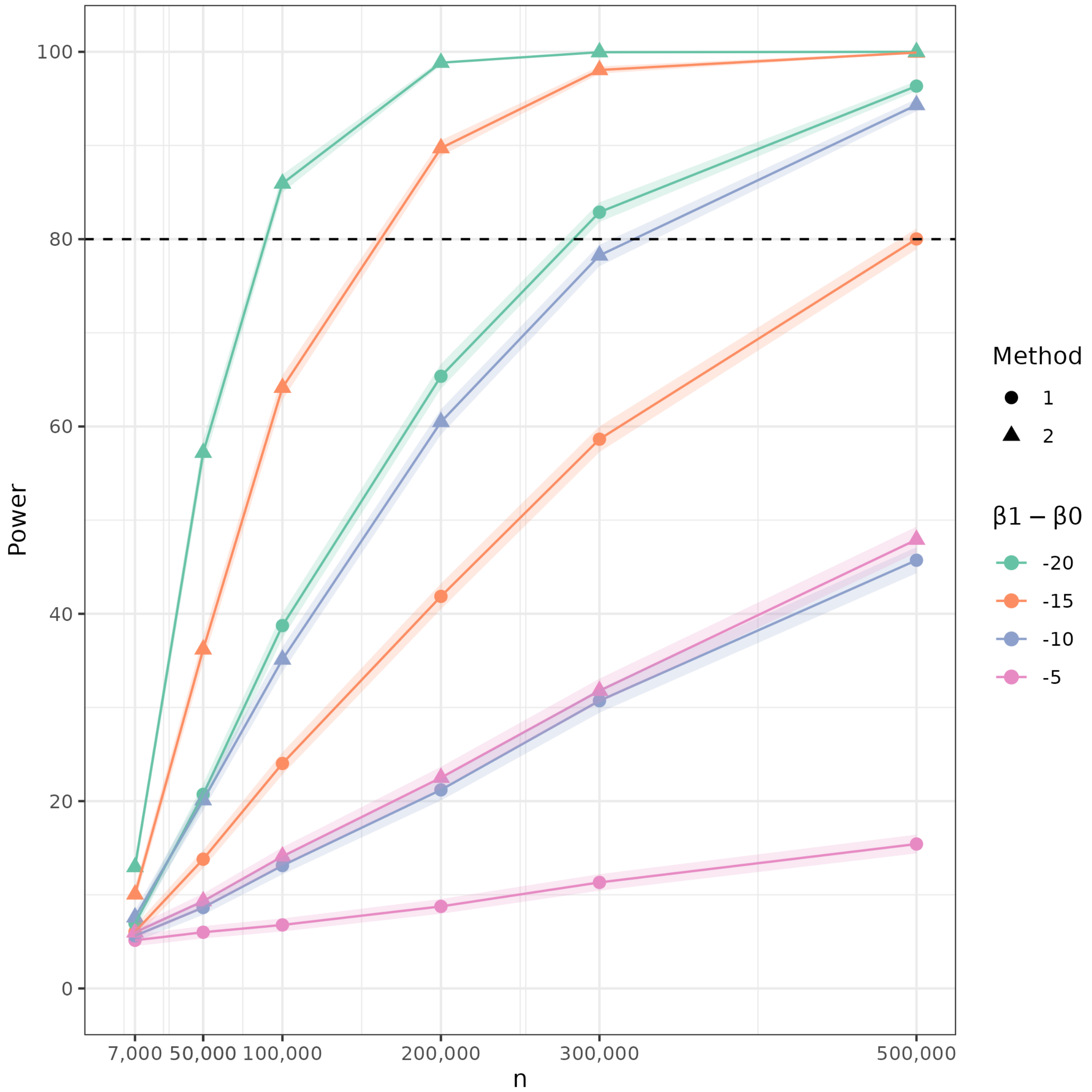

Figure S3: *Power to estimate $\beta_1 - \beta_0$ for different samples sizes (on the x-axis) applying method 1 and method 2 (see different shapes). The different colours refer to the different simulated data sets with changing $\beta_1 - \beta_0$ values.*

6 Appendix: Minor correction to "The Triangulation WIthin a STudy (TWIST) framework for causal inference within pharmacogenetic research" [3]

We noticed a mistake in the description of the conditions under which the Robust Genetically Moderated Treatment Effect (RGMTE) estimate is a consistent estimate for the true Genetically Moderated Treatment Effect, henceforth referred to as β_{GMTE}. In the following we describe this mistake and its implications for the TWIST framework.
Here, as in the original publication ([3]), let Y equal the outcome of interest, T be the binary treatment indicator, G be the binary pharmacogenetic variant that interacts with treatment, and U represents all confounders (i.e. common predictors of) T and Y. In the Supplementary Material (of Bowden et al. [3]) subsection titled "Consistency of the RGMTE estimate", it is shown (correctly) that the RMGTE(Y) estimand equals:

$$\begin{aligned}
&E[Y|T=1,G=1]-E[Y|T=1,G=0]-(E[Y|T=0,G=1]-E[Y|T=0,G=0]) \\
&=\beta_1-\beta_0+\gamma_{YU}(\{E[U|T=1,G=1]-E[U|T=1,G=0]\}-\{E[U|T=0,G=1]-E[U|T=0,G=0]\}) \\
&=\beta_1-\beta_0+\gamma_{YU}(\{\bar{U}_{11}-\bar{U}_{10}\}-\{\bar{U}_{01}-\bar{U}_{00}\}) \\
&=\beta_{GMTE}+\gamma_{YU}\beta_{RGMTE}(U) \\
&=\beta_{GMTE}+B, \qquad (1)
\end{aligned}$$

where the $\gamma_{.}$ and $\beta_{.}$ parameters' interpretation is shown in Figure S4 below, B represents the bias term and $\bar{U}_{ij}=E[U|T=i,G=j]$. Figure S4 formed the basis for the simulation study in Bowden et al. [3].

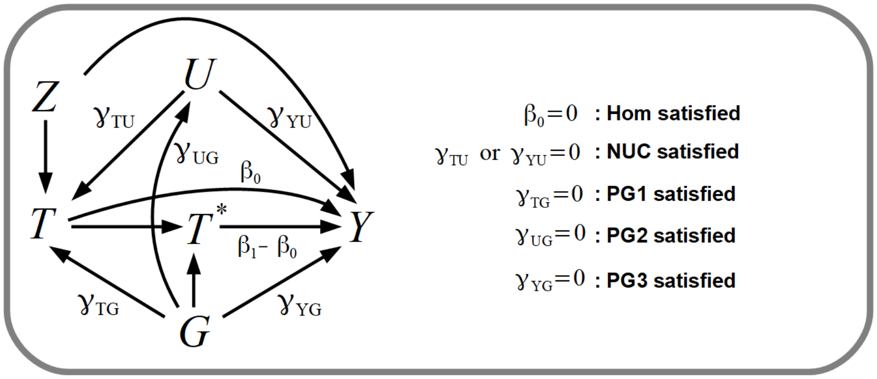

Figure S4: *Causal diagram with parameterised edges underlying the simulation study in our original publication.*

It was (correctly) concluded that the $RGMTE(Y)$ estimand is equivalent to β_{GMTE} if the $RGMTE(U)$ estimand is zero, and subsequently that

$$RGMTE(U)=0 \Longleftarrow Cov(U,G)|T=Cov(U,G). \qquad (2)$$

However, the conclusion about which assumptions are required to make the covariance between G and U independent of T, as in (2), were incorrect. Specifically, and with reference to Figure S4, if the parameters $\gamma_{TU}=0$ **or** (γ_{TG} **and** $\gamma_{UG}=0$), then covariance condition in (2) is satisfied hence the RGMTE(U) is zero. From equation (1), $B=0$ if either $\gamma_{YU}=0$ or if $\beta_{RGMTE}(U)=0$, hence the assumptions for the RGMTE(Y) to consistently target β_{GMTE} are:

- NUC $\cup$ (PG1 $\cap$ PG2).
- and **not** NUC $\cup$ PG1, as originally stated

Here, as in [3], NUC stands for 'No Unmeasured Confounders', PG1 stands for $G \perp\!\!\!\perp T|U$ and PG2 stands for $G \perp\!\!\!\perp U$ ($\perp\!\!\!\perp$ denoting statistical independence).

We now explain how we derived the correct assumption set and why this error was missed in the original simulation study. We will make the simplifying assumption that γ_{YU} is non-zero, meaning that there always unmeasured variables in an analysis that predict the outcome. In this case, there are four scenarios to consider:

(i) $\gamma_{TU} = 0$ and:

(a) $\gamma_{UG} = 0$, in which case $\bar{U}_{11} = \bar{U}_{10} = \bar{U}_{01} = \bar{U}_{00}$,
hence $Cov(U, G|T = 1) = Cov(U, G|T = 0) \implies \beta_{RGMTE}(U) = 0$

(b) $\gamma_{UG} \neq 0$, in which case $\bar{U}_{11} = \bar{U}_{01}$ and $\bar{U}_{10} = \bar{U}_{00}$,
hence $Cov(U, G|T = 1) = Cov(U, G|T = 0) \implies \beta_{RGMTE}(U) = 0$

(ii) $\gamma_{TU} \neq 0$ and :

(a) $\gamma_{UG} = 0$ and $\gamma_{TG} = 0$ in which case $\bar{U}_{11} = \bar{U}_{10}$ and $\bar{U}_{01} = \bar{U}_{00}$,
hence $Cov(U, G|T = 1) = Cov(U, G|T = 0) \implies \beta_{RGMTE}(U) = 0$

(b) $\gamma_{UG} \neq 0$ or $\gamma_{TG} \neq 0$ in which case $\bar{U}_{11} \neq \bar{U}_{10}$ and $\bar{U}_{01} \neq \bar{U}_{00}$,
hence $Cov(U, G|T = 1) \neq Cov(U, G|T = 0)$ and $\beta_{RGMTE}(U) \neq 0$.

Therefore we can conclude that the bias term $B = 0$ if

$$\gamma_{YU} = 0 \cup \gamma_{TU} = 0 \cup (\gamma_{UG} = 0 \cap \gamma_{TG} = 0)$$
$$\gamma_{YU} = 0 \cup \gamma_{TU} = 0 \cup (PG1 \cap PG2)$$
$$NUC \cup (PG1 \cap PG2)$$

A positive consequence of this mistake is that, if PG1 and PG2 hold, then G and T should be independent. Therefore, we now realise that it is possible to falsify the assumptions for unbiased estimation of $\beta_{GMTE}(Y)$ via the RGMTE method by testing for an association between G and T.

If the confounding is weak for example due to a low value of γ_{UG}, then the bias is small and therefore hard to detect. This was the reason that we did not detect the importance of PG2 assumption violation in the simulations in the original paper. We show in Scenario a) - d) in Table S8 how the bias becomes more obvious with increased confounding. The power to detect that G and T are not independent also increases. Scenario e) shows that if PG1 and PG2 are satisfied, the $RGMTE(Y) = \beta_{GMTE}$, even for large confounding due to a large value of γ_{TU}.

Scenario & Assumption(s) violated		Estimator		Power to detect T-G association (%)
		RGMTE(Y)	RGMTE(U)	
a) All except PG1 are violated: Scenario 6 in the original paper	Est	-0.496	0.0004	25
	S.E.	0.095	0.085	
	Coverage (%)	96		
b) All except PG1 are violated: Larger value of γ_{UG}	Est	-0.488	0.024	100
	S.E.	0.135	0.121	
	Coverage	96		
c) All except PG1 are violated: Larger value of γ_{TU}	Est	-0.469	0.055	100
	S.E.	0.074	0.061	
	Coverage	93		
d) All except PG1 are violated, larger values of γ_{UG} and γ_{TU}	Est	-0.226	0.514	100
	S.E.	0.295	0.247	
	Coverage	85		
e) PG1 and PG2 satisfied, large value of γ_{TU}	Est	-0.501	0.002	4
	S.E.	0.068	0.055	
	Coverage	95		

Table S8: Simulation results showing why Scenario 6 in Bowden et al. [3] appeared unbiased. a) Original simulation study parameters. b) - d) modified parameter values that accentuate the bias. e) PG1 and PG2 are satisfied and despite large confounding, the RGMTE(Y) is unbiased. The results are unbiased (or the bias is very small) if the power to detect an association between G and T is low.In each case the true value of the RGMTE(Y) is $\beta_1 - \beta_0 = -0.5$. The simulation was performed with a sample size of $n = 1000$ and was repeated $N = 500$ times.

References

[1] Angrist, Joshua D., Imbens, Guido W., and Rubin, Donald B. "Identification of Causal Effects Using Instrumental Variables". In: *Journal of the American Statistical Association* 91.434 (1996). ISSN: 1537274X. DOI: 10.1080/01621459.1996.10476902.

[2] Bowden, Jack et al. "Connecting Instrumental Variable methods for causal inference to the Estimand Framework". In: *Statistics in Medicine* 40.25 (2021), pp. 5605–5627. ISSN: 10970258. DOI: 10.1002/SIM.9143.

[3] Bowden, Jack et al. "The Triangulation WIthin a STudy (TWIST) framework for causal inference within pharmacogenetic research". In: *PLoS Genetics* 17.9 (2021). ISSN: 15537404. DOI: 10.1371/JOURNAL.PGEN.1009783.
